# Effects of positive psychological interventions on quality of life in patients with first-episode depression

**DOI:** 10.3389/fpsyt.2025.1564225

**Published:** 2025-07-25

**Authors:** Lizhao Lv, Keyan Han, Limin Meng, Jincheng Wang, Hui Yin, Kenan Ren, Zhihua Liu

**Affiliations:** Department of Psychiatry, The First Hospital of Hebei Medical University, Shijiazhuang, Hebei, China

**Keywords:** positive psychological intervention, improvement, first-episode depression, quality of life, depression status, psychological resilience scores, self-acceptance

## Abstract

**Objective:**

This study aims to investigate the effects of positive psychological interventions (PPIs) on improving the quality of life in first-episode depression patients.

**Methods:**

A total of 200 first-episode depression patients were randomly assigned to a control group (n=100, conventional nursing) or a study group (n=100, conventional care plus four weekly 60–80-minute PPI sessions over one month). Depression severity (Hamilton Depression Rating Scale, HAMD), psychological resilience (Connor-Davidson Resilience Scale, CD-RISC), self-acceptance (Self Acceptance Questionnaire, SAQ), and quality of life (SF-36) were assessed pre-intervention and 3 months post-intervention.

**Results:**

Baseline measures showed no significant intergroup differences (P>0.05). At 3 months, the study group exhibited greater HAMD reduction (Δ=11.16 vs. Δ=9.09 in controls; mean post-intervention: 17.03 ± 3.45 vs. 19.23 ± 3.21, p<0.001), higher CD-RISC improvement (Δ=36.27 vs. Δ=29.54; 80.09 ± 7.86 vs. 73.92 ± 7.36, p<0.001), and increased SAQ total scores (Δ=18.17 vs. Δ=12.25; 43.47 ± 9.21 vs. 37.84 ± 8.24, p<0.001). SF-36 total scores improved by 52.0% in the study group (540.41 ± 32.66 vs. 276.41 ± 27.99) compared to 42.5% in controls (487.85 ± 31.89 vs. 279.48 ± 26.39, p<0.001).

**Conclusion:**

PPIs delivered over four weeks significantly enhance quality of life and psychological outcomes in first-episode depression, supporting clinical adoption.

## Introduction

Depression is a serious mental disorder that falls under the category of mood disorders, characterized by high incidence and recurrence rates, as well as a significant prevalence among the population (1). According to the World Health Organization (WHO), approximately 350 million people globally suffer from depressive disorders. In a mental health survey conducted across 17 countries, it was found that one in every 20 individuals had experienced or was currently experiencing depression, with an annual prevalence rate of 1.5% and a lifetime prevalence of 3.1%. Notably, reports indicate that up to one-fifth of women may experience depressive symptoms postpartum (2, 3).

As the pressures of modern life continue to escalate, the incidence of depression is rising annually. Negative emotions can lead patients to exhibit aggressive behaviors towards others or self-harm, and in severe cases, there may be a significant risk of suicide, all of which severely diminish the quality of life for these individuals (4, 5). Researchers have suggested that enhancing nursing care interventions for first-episode depression patients can effectively address existing psychological issues and provide adequate emotional support, ultimately alleviating depressive symptoms and improving overall quality of life (6).

Historically, clinical interventions have included some form of psychological support, but these interventions often lack specificity and fail to achieve the desired therapeutic outcomes (7). Positive psychology, a concept introduced by Professor Martin Seligman at the University of Pennsylvania in 2000, goes beyond merely alleviating negative emotions. It focuses on identifying and utilizing individuals’ positive strengths and qualities to maximize their potential for achieving a fulfilling life (8). Notably, while PPIs have been widely studied in chronic or recurrent depression populations, their application in first-episode cases remains underexplored despite evidence suggesting that early-stage interventions may prevent symptom chronicity and optimize neuroplasticity-driven recovery (9).

With advancements in techniques and the accumulation of clinical experience, the adoption of positive psychological intervention models has begun in healthcare settings (10). Recent systematic reviews further validate that multi-component psychological strategies, such as cognitive-behavioral techniques combined with social support, effectively reduce depressive symptomatology and enhance quality of life, even in populations distinct from clinical depression patients (e.g., caregivers of dependent elderly individuals) (11). Preventive trials in early-stage depression further indicate that strength-based interventions delivered during initial episodes correlate with higher remission rates and sustained psychosocial functioning compared to standard therapies (12). Implementing these interventions can effectively transform patients’ negative mental states, foster a positive mindset, and consequently enhance their cooperation and quality of life (10). Therefore, this study aims to explore the application effects of positive psychological interventions in improving the quality of life of first-episode depression patients. We hypothesized that PPIs would significantly improve quality of life, psychological resilience, and self-acceptance in first-episode depression patients compared to conventional care.

## Materials and methods

### General information

A total of 200 patients with first-episode depression admitted to our hospital from March 2023 to March 2024 were selected as subjects for this study. These patients were randomly divided into a study group and a control group using a computer-generated randomization sequence (block randomization with a 1:1 allocation ratio) prepared by an independent statistician. Opaque sealed envelopes containing group assignments were opened sequentially after baseline assessments to ensure allocation concealment, with 100 cases in each group. Inclusion criteria: (1) Diagnosed with depression according to the diagnostic criteria of the “Revised Guidelines for the Prevention and Treatment of Depression” (13); (2) first episode of the illness; (3) Hamilton Depression Rating Scale (HAMD) (14) score > 7; (4) age ≥ 18 years; (5) duration of illness ≥ 1 month; (6) education level of primary school or above; (7) informed consent obtained from all patients’ families. Exclusion criteria: (1) severe physical diseases; (2) comorbid other psychiatric disorders; (3) presence of other acute or chronic severe diseases; (4) pregnancy or lactation; (5) recent major life events, such as natural disasters, loss of loved ones, etc.; (6) history of cerebrovascular disease, head trauma, or intellectual disabilities; (7) participation in other studies; (8) history of non-steroidal anti-inflammatory drugs or antidepressant use within one week prior to inclusion; (9) evident suicidal tendencies. *A priori* power analysis (G*Power 3.1) determined the sample size (15). Assuming a medium effect size (d = 0.5), α = 0.05, and 80% power for detecting group differences in HAMD scores, the required sample was 128 (64 per group). We recruited 200 patients to account for a potential 20% attrition rate.

### Participant flow

**Figure 1:**
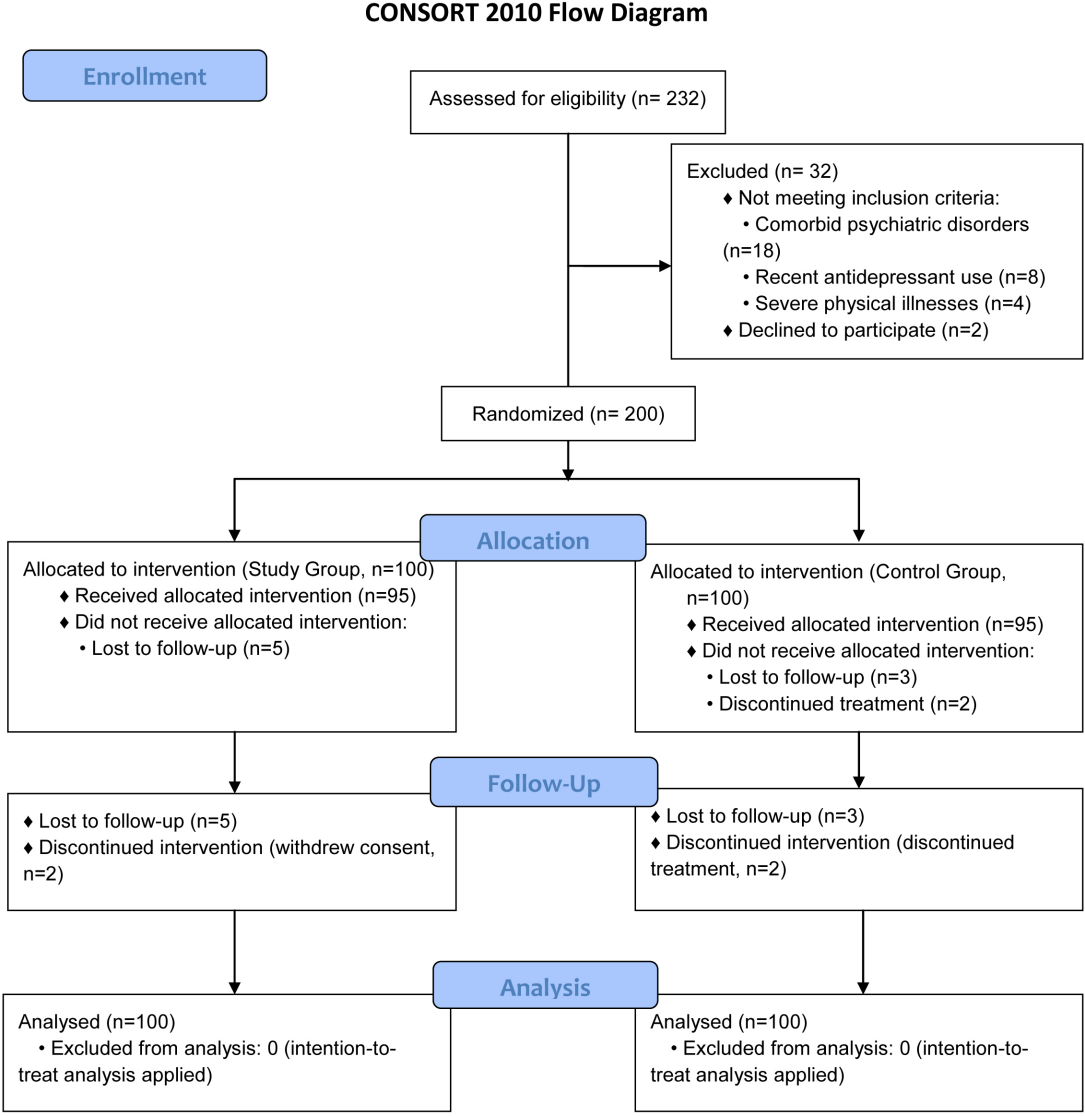
The participant flow diagram. Initially, 232 patients with first-episode depression were screened. Thirty-two were excluded (18 due to comorbid psychiatric disorders, 8 for recent antidepressant use, 4 with severe physical illnesses, 2 declining participation). The remaining 200 were randomized (100 study, 100 control). During the 3-month intervention, 12 participants dropped out (study group: 5 lost to follow-up, 2 withdrew consent; control group: 3 lost to follow-up, 2 discontinued treatment.

**Figure 1** illustrates the participant flow diagram. Initially, 232 patients with first-episode depression were screened for eligibility. Thirty-two were excluded (18 due to comorbid psychiatric disorders, 8 for recent antidepressant use, 4 with severe physical illnesses, and 2 declining participation). The remaining 200 were randomized into study (n=100) and control (n=100) groups. During the 3-month intervention, 12 participants (6%) dropped out (study group: 5 lost to follow-up, 2 withdrew consent; control group: 3 lost to follow-up, 2 discontinued treatment). Missing data were addressed via intention-to-treat analysis with multiple imputation.

### Methods

The control group received routine nursing interventions, including weekly 60–80 minute sessions of medication guidance (15–20 minutes on dosage adjustment and side effects), safety care (10–15 minutes on risk monitoring), psychological intervention (15–20 minutes of general supportive counseling), disease education (10–15 minutes on depression pathophysiology), and daily living care (10–15 minutes on sleep hygiene).

In addition to the routine care, the study group received a standardized 4-stage positive psychological intervention (PPI) developed by our team, with standardized training for psychologists and nurses (8-hour workshop) once a week (on Saturdays or Sundays) for 60–80 minutes per session, spanning a total of four stages conducted by a trained and qualified psychologist and professional nurse under weekly supervision by a senior clinical psychologist. Treatment fidelity was monitored through session checklists (completed by interveners) and random audits of 20% of session recordings by an independent evaluator, with >90% adherence to protocol components. Outcome assessors (trained research assistants uninvolved in intervention delivery) were blinded to group allocation; participants were instructed not to discuss treatment details during assessments to maintain blinding integrity.

- **Stage 1 (Strengths Identification)**: In a quiet, private therapy room, patients were given paper and pens with words such as confident, brave, steady, generous, cheerful, strong, resilient, helpful, wise, kind, and frugal. Patients and their families were instructed to select five words that represented their strengths. Patients were encouraged to describe their recognized strengths, with family members able to contribute, fostering a collaborative atmosphere for self-recognition. Patients were also encouraged to write about events that reflect their strengths to share in the next session.
- **Stage 2 (Positive Sharing)**: Patients were guided to share pleasant life and study experiences to engage in positive sharing activities. Discussions on “joyful living” were conducted using sharing, narration, and summarization methods, allowing patients to fully experience joy and adopt an open, constructive attitude toward life. After discussion, patients recorded “three joyful things” in their diaries for the next week and summarized why these were joyful, encouraging them to share recent pleasant experiences in the next intervention.
- **Stage 3 (Emotional Expression)**: Patients were encouraged and helped to express negative emotions in a rational and scientific manner. They shared past unpleasant emotional experiences such as sadness, despair, fear, and helplessness, along with correct coping strategies. Patients were instructed to write about unpleasant events and encouraged to express feelings through talking or journaling, avoiding self-harmful behaviors. In the next session, they were encouraged to explain their emotional management strategies when facing unpleasant events.
- **Stage 4 (Life Savoring)**: Patients were guided to savor life’s pleasures and enjoy joyful feelings, reflecting on beautiful moments. They were encouraged to actively describe their ideal life and how they would define their behaviors, character, and achievements, recording these details. Patients were taught to reflect on their behaviors, control emotions and inappropriate actions, and achieve their ideal goals.

The PPI protocol was standardized (fixed stages, duration, and core exercises) but allowed personalized application (e.g., strength words selected individually, self-defined joyful events). Therapists followed a manual but could adjust phrasing to enhance participant engagement (e.g., rephrasing questions for clarity).

### Observational indicators

#### Depressive symptoms

Before the intervention (1 day prior) and 3 months after the intervention, depression was assessed using the Hamilton Depression Rating Scale (HAMD). This scale consists of 17 items, with a higher total score indicating more severe depressive symptoms; scores of 7–17 indicate mild depression, 18–24 indicate moderate depression, and a score of ≥25 indicates severe depression. HAMD demonstrated good reliability in the current sample (Cronbach’s α = 0.84).

#### Psychological resilience score

Psychological resilience was evaluated using the Connor-Davidson Resilience Scale (CD-RISC) (16) before the intervention (1 day prior) and 3 months after the intervention. This scale includes 25 dimensions to assess patients’ psychological resilience, using a 5-point Likert scale where each item is scored from 0 to 4. The scoring is as follows: 0 points - completely incorrect; 1 point - rarely correct; 2 points - sometimes correct; 3 points - usually correct; 4 points - almost always correct. The maximum score is 100, with higher scores indicating stronger psychological resilience. CD-RISC showed excellent internal consistency (Cronbach’s α = 0.91).

#### Self-acceptance level

Self-acceptance was surveyed using the Self Acceptance Questionnaire (SAQ) (17) before the intervention (1 day prior) and 3 months after the intervention. This scale includes two factors: self-evaluation and self-acceptance, each comprising 8 items for a total of 16 items. Each item is scored using a 4-point scale: 1 - very similar; 2 - basically similar; 3 - basically opposite; 4 - very opposite. The total score for each factor is the sum of the scores for the 8 items, and the total score for the scale is the sum of the scores from both factors, ranging from 16 to 64. Higher scores indicate better self-acceptance. SAQ reliability was confirmed (Cronbach’s α = 0.88).

#### Quality of Life

Quality of life was assessed using the 36-Item Short Form Health Survey (SF-36) (18) before the intervention (1 day prior) and 3 months after the intervention. This scale evaluates various aspects including physical functioning, role limitations due to physical health, social functioning, role limitations due to emotional problems, bodily pain, general health perception, vitality, and mental health. Each item is scored from 0 to 100, with higher scores indicating better quality of life. SF-36 scale exhibited high reliability (Cronbach’s α = 0.89).

### Statistical analysis

Statistical analysis was performed using SPSS 26.0. Intention-to-treat analysis with multiple imputation (Fully Conditional Specification method, 5 imputations) was applied for participants with missing data (n = 12, 6%). Sensitivity analyses comparing complete cases and imputed datasets showed no significant differences in outcomes. For normally distributed and homogeneously variance continuous data, results are presented as mean ± standard deviation. Paired t-tests were used for inter-group comparisons, and independent t-tests were used for intra-group comparisons. Categorical data are presented as rates (%), and comparisons were made using the Chi-square (χ^2^) test. A p-value of <0.05 was considered statistically significant.

## Results

### Comparison of general information between two groups

The demographic characteristics, including gender, age, duration of illness, and severity of condition, were compared between the two groups. No statistically significant differences were found (P > 0.05), as shown in **Table 1**.

**Table 1:** Comparison of general information between two groups.

Item	Control Group (n=100)	Study Group (n=100)	t/χ²	P
Gender (n, %)	61 (61%)/39 (39%)	67 (67%)/33 (33%)	0.375	0.540
Age (years)	30.23 ± 5.28 (18–50)	30.09 ± 5.67 (19–52)	-0.181	0.857
Duration (months)	3.63 ± 0.76 (2–6)	3.74 ± 0.81 (2–7)	0.990	0.323
Severity (n, %)			0.375	0.829
- Mild	32 (32%)	30 (30%)		
- Moderate	39 (39%)	37 (37%)		
- Severe	29 (29%)	33 (33%)		
Education Level (n, %)			0.015	0.903
- Bachelor’s/Diploma	66 (66%)	71 (71%)		
- High School/Below	34 (34%)	29 (29%)		
Residence (n, %)			0.676	0.411
- Rural	78 (78%)	73 (73%)		

### Comparison of depression levels between two groups

Before the intervention (1 day prior), the depression levels of the two groups showed no statistically significant difference (P > 0.05). However, after 3 months of intervention, the study group exhibited significantly lower depression scores compared to the control group (P < 0.05), as detailed in **Table 2**.

**Table 2:** Comparison of depression levels between two groups (mean±sd, scores).

Time	Control Group (n=100)	Study Group (n=100)	t	P
Pre-intervention (1d)	28.32 ± 4.32	28.19 ± 4.87	-0.200	0.842
Post-intervention (3 months)	19.23 ± 3.21	17.03 ± 3.45	-4.669	<0.001

### Comparison of psychological resilience scores between two groups

The psychological resilience scores of both groups prior to the intervention showed no statistically significant difference (P > 0.05). After 3 months of intervention, the study group demonstrated a significantly higher psychological resilience score compared to the control group (P < 0.05), as presented in **Table 3**.

**Table 3:** Comparison of psychological resilience scores between two groups (mean±sd, scores).

Time	Control Group (n=100)	Study Group (n=100)	t	P
Pre-intervention (1d)	44.38 ± 6.83	43.82 ± 7.32	-0.559	0.577
Post-intervention (3 months)	73.92 ± 7.36	80.09 ± 7.86	5.730	<0.001

### Comparison of self-acceptance between two groups

Before the intervention (1 day prior), there was no statistically significant difference in self-evaluation, self-acceptance, and total scores between the two groups (P > 0.05). However, after 3 months of intervention, the research group showed higher scores in self-evaluation, self-acceptance, and total scores compared to the control group, with significant differences (P < 0.05), as shown in **Table 4**.

**Table 4:** Comparison of self-acceptance between two groups (mean±sd, points).

Item	Time	Control Group (n=100)	Research Group (n=100)	t	P
Self-Evaluation	Pre-intervention 1d	12.76 ± 3.72	12.54 ± 3.97	-0.404	0.686
	Post-intervention 3 months	18.08 ± 4.09	21.13 ± 4.27	5.158	<0.001
Self-Acceptance	Pre-intervention 1d	12.83 ± 3.86	12.76 ± 4.03	-0.125	0.900
	Post-intervention 3 months	19.76 ± 4.15	22.34 ± 4.94	3.999	0.001
Total Score	Pre-intervention 1d	25.59 ± 7.58	25.30 ± 8.00	-0.263	0.793
	Post-intervention 3 months	37.84 ± 8.24	43.47 ± 9.21	4.556	<0.001

### Comparison of quality of life between two groups

Before the intervention (1 day prior), there was no statistically significant difference in physical function, physical role, social function, emotional role, bodily pain, overall health status, vitality, mental health, and total scores between the two groups (P > 0.05). However, after 3 months of intervention, the research group showed higher scores in all these areas compared to the control group, with significant differences (P < 0.05), as shown in **Table 5**.

**Table 5:** Comparison of quality of life between two groups (mean±sd, points).

Item	Time	Control Group (n=100)	Study Group (n=100)	t-value	P-value
Physical Function	Pre-Intervention	41.28 ± 3.29	41.21 ± 3.41	-0.148	0.883
	Post-Intervention	63.76 ± 3.43	70.09 ± 4.05	11.927	<0.001
Physical Role	Pre-Intervention	42.07 ± 3.14	41.87 ± 3.09	-0.454	0.650
	Post-Intervention	60.23 ± 3.49	68.34 ± 3.76	–	<0.001
Social Function	Pre-Intervention	41.65 ± 3.18	41.23 ± 3.27	-0.921	0.358
	Post-Intervention	60.08 ± 3.87	65.37 ± 3.51	10.125	<0.001
Emotional Role	Pre-Intervention	31.78 ± 3.21	31.09 ± 3.48	-1.457	0.147
	Post-Intervention	62.02 ± 3.76	68.94 ± 3.82	12.910	<0.001
Overall Health Status	Pre-Intervention	31.09 ± 3.17	30.94 ± 3.45	-0.320	0.749
	Post-Intervention	61.33 ± 3.81	65.49 ± 3.92	7.610	<0.001
Vitality	Pre-Intervention	30.78 ± 3.46	30.12 ± 3.51	-1.339	0.182
	Post-Intervention	60.07 ± 4.53	66.83 ± 4.03	11.149	<0.001
Mental Health	Pre-Intervention	30.56 ± 3.51	30.02 ± 3.87	-1.034	0.303
	Post-Intervention	60.34 ± 4.87	67.03 ± 4.76	9.824	<0.001
Bodily Pain	Pre-Intervention	30.27 ± 3.43	29.93 ± 3.91	-0.654	0.514
	Post-Intervention	60.02 ± 4.13	68.32 ± 4.81	13.092	<0.001
Total Score	Pre-Intervention	279.48 ± 26.39	276.41 ± 27.99	-0.798	0.426
	Post-Intervention	487.85 ± 31.89	540.41 ± 32.66	11.514	<0.001

## Discussion

First-episode depression is a typical depressive disorder with complex etiology, primarily related to various social, environmental, and psychological factors. The onset of this condition is closely associated with psychological factors, and relying solely on medication can only provide limited relief for depressive symptoms, making it difficult to achieve a cure. Therefore, effective nursing strategies are necessary to alleviate the psychological distress of patients with depression and improve their quality of life (19). Positive psychology posits that positive behaviors and cognitions can, to some extent, mitigate psychological suffering and enhance well-being. Positive psychological interventions primarily use the positive psychology model as a theoretical framework, emphasizing that it is not sufficient to merely instruct patients to maintain happiness and positive behaviors to attain well-being. Instead, these interventions aim to alleviate negative emotions through positive psychology and positive behaviors (8). Currently, this approach has been incorporated into the treatment of various mental disorders and has been proven effective.

Research by Yao et al. (20) indicates that effective nursing interventions can alleviate depression in patients with first-episode depression. Serrano-Ripoll and colleagues (21) found that targeted nursing interventions for patients with depression can moderately reduce depressive symptoms. Hernandez et al. (22) reported that positive psychological interventions can effectively improve depressive symptoms in patients with depression, demonstrating significant efficacy. The results of this study show that after three months of intervention, the depression levels in the experimental group were lower than those in the control group (P < 0.05), which is consistent with the aforementioned studies and suggests that positive psychological interventions can effectively improve the depressive conditions of patients with first-episode depression. This aligns with Fredrickson’s Broaden-and-Build Theory (23), where cultivating positive emotions through strength identification broadens attentional focus and builds enduring psychological resources (e.g., resilience), thereby disrupting the negative cognitive narrowing typical of depression. This may be due to the fact that positive psychological interventions guide patients to recognize their strengths, enabling them to better appreciate their own attributes. Implementing positive psychological interventions allows patients to maintain a positive attitude, enhance their hope and confidence, mobilize their potential, and alleviate suffering in their lives, leading to significant improvement in depressive symptoms.

With the rise and ongoing development of positive psychology, researchers have begun to explore suicidal ideation from a new perspective, emphasizing the development and exploration of individual potential such as psychological resilience (24). Psychological resilience is a potential universally possessed by individuals, referring to the capacity to adapt successfully and develop positively when facing significant life stressors such as disasters, trauma, adversity, or threats (25). Research by Taylor (24) suggests that increasing the psychological resilience of patients with depression or reducing cognitive fusion can alleviate anxiety and depression. Danand colleagues (26) found that mental health interventions can enhance the psychological resilience of patients with depression, which may be beneficial for their recovery process. The results of this study show that after three months of intervention, the psychological resilience scores of the experimental group were higher than those of the control group (P < 0.05), indicating that positive psychological interventions can effectively enhance the psychological resilience of patients with first-episode depression. From a cognitive-behavioral perspective (27), PPIs may counteract depressive cognitive fusion (e.g., overidentification with negative thoughts) by reinforcing adaptive self-narratives, thereby increasing emotional flexibility—a key resilience mechanism. This may be because positive psychological interventions guide patients to share pleasant events from their lives and studies, allowing them to fully experience joy and maintain a positive mood, adopt an open and correct attitude towards life, and promote a proactive approach to life, which in turn improves depressive symptoms, stabilizes emotions, continuously enhances cognitive levels, and strengthens psychological resilience.

Self-acceptance is an important component of individual social adaptability; the lower the level of self-acceptance, the poorer the social adaptability of the individual (28). Research indicates that the level of self-acceptance in patients with depression affects their adaptability to life and their confidence in disease recovery, and improving the self-acceptance of patients has a positive effect on preventing suicide among patients (28). Studies have shown that self-role identity care for patients with mental illness helps them better understand themselves, enabling them to adapt more effectively to changes in their roles after becoming ill (29). Research by Berman et al. (30) indicates that effective nursing interventions can enhance the level of self-acceptance in patients with depression and alleviate clinical symptoms. Liu et al. (31) concluded that positive psychological interventions can significantly improve the depressive symptoms and self-acceptance levels in patients with depression. The results of this study show that after three months of intervention, the self-evaluation, self-acceptance, and total scores of the study group were higher than those of the control group (P < 0.05), suggesting that positive psychological interventions can effectively enhance the self-acceptance of patients with first-episode depression. Beck’s model (32) posits that depression perpetuates through distorted self-schemas; PPIs’ emphasis on strength recognition may facilitate cognitive restructuring by challenging maladaptive self-perceptions, thus improving acceptance. This may be because positive psychological interventions encourage and assist patients in rationally and scientifically expressing negative emotions, freeing themselves from the distress of depression, helping them build confidence in treatment, ensuring affirmation of self-worth, and ultimately achieving the goal of enhancing self-acceptance.

Depression is a severe psychological disorder characterized by low self-evaluation, guilt, negativity, and even tendencies toward self-harm and suicide, which seriously affects the happiness of the individuals and their families and decreases the quality of life (1). Research by Kolovos et al. (33) indicates that effective psychological interventions positively influence the quality of life of patients with depression. Craig et al. (34) pointed out that effective nursing interventions can improve the quality of life of patients with depression. McPherson et al. (35) emphasized the potential benefits of psychotherapy in improving the quality of life and functioning of patients with persistent depression. The results of this study show that after three months of intervention, the study group had higher scores in physical functioning, role limitations due to physical health, role limitations due to emotional problems, bodily pain, general health, vitality, mental health, and total score compared to the control group (P < 0.05), suggesting that positive psychological interventions can effectively enhance the quality of life of patients with first-episode depression. Notably, while Hernandez et al. (22) reported comparable quality-of-life improvements in mixed depression cohorts, our first-episode cohort exhibited faster gains in vitality (ΔSF-36 vitality = 18.3 vs. Δ = 12.1 in Hernandez), possibly reflecting greater neurocognitive responsiveness to early intervention (36). This may be due to positive psychological interventions guiding patients to savor the joys of life and enjoy pleasant feelings, helping them recall beautiful moments, and maintaining a positive, high-spirited, and optimistic mindset throughout the nursing process and in future life, thereby enhancing their sense of happiness and improving their quality of life.

Furthermore, while our study focused on first-episode depression, recent national consensus guidelines have emphasized the importance of early-stage, individualized interventions as part of a broader stepped-care model for depression management, including treatment-resistant depression (TRD). In this regard, Maina et al. (37) provided a Delphi-based consensus on TRD management in Italy, underscoring the value of patient-centered, multimodal approaches that align with the principles underlying positive psychological interventions used in this study.

In summary, positive psychological interventions can effectively improve the depressive condition of patients with first-episode depression, enhance psychological resilience, self-acceptance, and quality of life, and promote patient recovery. However, this study has some limitations, including being a single-center study (risk of selection bias), the presence of certain subjectivity in the scales used (self-report measures may inflate social desirability effects), a relatively short intervention duration (3 months), and lack of comparison to active treatments (e.g., CBT). Future research should explore moderators of PPI efficacy (e.g., age, baseline severity, education level) to identify subgroups that benefit most from this intervention. Multicenter trials with blinded assessments, objective biomarkers (e.g., cortisol levels, fMRI for neural plasticity), and extended follow-ups to evaluate relapse prevention are warranted.

## Data Availability

The original contributions presented in the study are included in the article/supplementary material. Further inquiries can be directed to the corresponding author.
